# Evaluation of conventional laparoscopic versus robot-assisted laparoscopic redo hiatal hernia and antireflux surgery: a cohort study

**DOI:** 10.1007/s11701-016-0558-z

**Published:** 2016-01-25

**Authors:** Robert C. Tolboom, Werner A. Draaisma, Ivo A. M. J. Broeders

**Affiliations:** Department of Surgery, Meander Medical Center, P.O. box 1502, 3800 BM Amersfoort, The Netherlands; Robotics and Minimal Invasive Surgery, University of Twente, Enschede, The Netherlands

**Keywords:** Gastroesophageal reflux, Fundoplication, Robotic surgical procedures, Da Vinci, Redo surgery

## Abstract

Surgery for refractory gastroesophageal reflux disease (GERD) and hiatal hernia leads to recurrence or persisting dysphagia in a minority of patients. Redo antireflux surgery in GERD and hiatal hernia is known for higher morbidity and mortality. This study aims to evaluate conventional versus robot-assisted laparoscopic redo antireflux surgery, with the objective to detect possible advantages for the robot-assisted approach. A single institute cohort of 75 patients who underwent either conventional laparoscopic or robot-assisted laparoscopic redo surgery for recurrent GERD or severe dysphagia between 2008 and 2013 were included in the study. Baseline characteristics, symptoms, medical history, procedural data, hospital stay, complications and outcome were prospectively gathered. The main indications for redo surgery were dysphagia, pyrosis or a combination of both in combination with a proven anatomic abnormality. The mean time to redo surgery was 1.9 and 2.0 years after primary surgery for the conventional and robot-assisted groups, respectively. The number of conversions was lower in the robot-assisted group compared to conventional laparoscopy (1/45 vs. 5/30, *p* = 0.035) despite a higher proportion of patients with previous surgery by laparotomy (9/45 vs. 1/30, *p* = 0.038). Median hospital stay was reduced by 1 day (3 vs. 4, *p* = 0.042). There were no differences in mortality, complications or outcome. Robotic support, when available, can be regarded beneficial in redo surgery for GERD and hiatal hernia. Results of this observational study suggest technical feasibility for minimal-invasive robot-assisted redo surgery after open primary antireflux surgery, a reduced number of conversions and shorter hospital stay.

## Introduction

Patients with refractory gastroesophageal reflux disease (GERD) or hiatal hernia may be offered surgical repair to alleviate their complaints. Antireflux surgery has a satisfactory outcome in 85–96 % of patients [1–4]. The remaining patients may experience persisting reflux symptoms, recurrence of GERD or suffer from severe dysphagia after surgery [4, 5].

Redo hiatal hernia and antireflux surgery mostly takes place in the first years after initial surgery with 1, 5 and 10-year cumulative reoperation rates of 1.7, 5.2 and 6.9 %, respectively [6].

Redo hiatal hernia and antireflux surgery is known for its technical difficulties. It has a higher mortality rate of up to 1 %, a higher intraoperative complication rate and a less satisfactory symptomatic outcome than primary antireflux surgery (ARS) [5, 7–11].

Many different surgical options exist to treat recurrent symptoms or severe dysphagia after primary reflux surgery. A literature review from the Netherlands has shown that currently most redo surgery is performed using open surgery via the abdominal route (34.7 %) or via thoracotomy (22.7 %). The minority of redo surgery is performed using the minimal invasive laparoscopic approach (36.3 %) [12]. While redo antireflux surgery is traditionally performed via open surgery, evidence exists of equality if not superiority of the minimally invasive laparoscopic approach [10, 13].

Laparoscopic redo surgery is usually limited to a 2-dimensional view. This hampers recognition of anatomic structures in areas with abundant adhesions or anatomical abnormalities while dissection at the GE-junction requires refined instrument control. In recent years, robot-assisted laparoscopic surgery has become more widely available to surgeons. Robotic systems contain 3D vision and camera control by the surgeon. They are especially useful for delicate dissections and offer benefits when suturing in relatively small, confined spaces due to its instruments that can mimic wrist-like motions.

Few comparative studies have been conducted to evaluate possible benefits of a robot-assisted laparoscopy over traditional laparoscopy for reflux disease [14–16]. While no additional value of the robotics system for primary ARS could be proven, the use of the Da Vinci robot may be beneficial for minimally invasive redo hiatal hernia and antireflux procedures due to their challenging nature.

This study aims to evaluate the results of a single surgeon with extensive laparoscopic and previous robotic experience, comparing conventional laparoscopic versus robot-assisted laparoscopic redo hiatal hernia and antireflux surgery, with the objective to detect possible advantages for the robot-assisted approach.

## Methods

The medical records of a cohort of consecutive adult patients referred to our center with persisting reflux symptoms, recurrence or severe dysphagia after previous antireflux surgery between 2008 and 2013 were analyzed. All patients were operated by the same surgeon, who had an >500 case experience in traditional, laparoscopic and robot-assisted surgery for reflux and hiatal hernia in a previous institute between 1998 and 2008.

The Da Vinci robot was introduced to our hospital in March 2011. Redo procedures before that date were performed using mostly conventional laparoscopy or in case of major previous abdominal surgery, by laparotomy. After the introduction of the robotic system, we endeavored to do all redo hiatal hernia and antireflux surgery robotically assisted.

Patient demographics, comorbidity, history of previous operations, pre- and postoperative symptoms, operative course, perioperative outcomes and follow-up data were gathered into a database.

### Preoperative workup

All patients were evaluated with a detailed history. Inquiries were made regarding their symptoms, outcomes after primary surgery and timing of symptom occurrence. All patients underwent esophagogastroduodenoscopy and either a CT-scan of chest and abdomen or barium swallow studies. A 24-h pH monitoring, esophageal manometry or gastric emptying studies were performed if necessary. Anatomical abnormalities in combination with symptoms were deemed essential to propose surgical reintervention.

### Surgical technique

All patients underwent dissection of adhesions, takedown of the previous fundoplication and adequate mobilization of the esophagus until sufficient intra-abdominal length was obtained. Next, posterior crural repair or release was performed as needed and a fundoplication was created. Polypropylene mesh was used at the site of the cruroplasty in most patients. Mesh was used increasingly in the course of time in line with growing experience and an increasing number of reports in literature. The choice for fundoplication was subject to the indication for the revision of the fundoplication. In case of severe preoperative dysphagia, 180° anterior fundoplication (Dor) was constructed. In case of persistent or recurrent GERD, a posterior 270° fundoplication (Toupet) was performed.

### Follow-up

Patients were followed in our outpatient clinic 6 weeks after surgery, after that only when needed. The most recent visit was used to calculate follow-up time.

### Statistical analysis

Data were analyzed using IBM^®^ SPSS^®^ Statistics 22. The results are presented as mean (95 % clearance interval) or median (interquartile range) or *n* (%). The Fischer’s exact test was used to compare categorical data. Continuous, unpaired data were compared using the Mann–Whitney *U* test. *P* < 0.05 was considered to be significant.

## Results

**Fig. 1:**
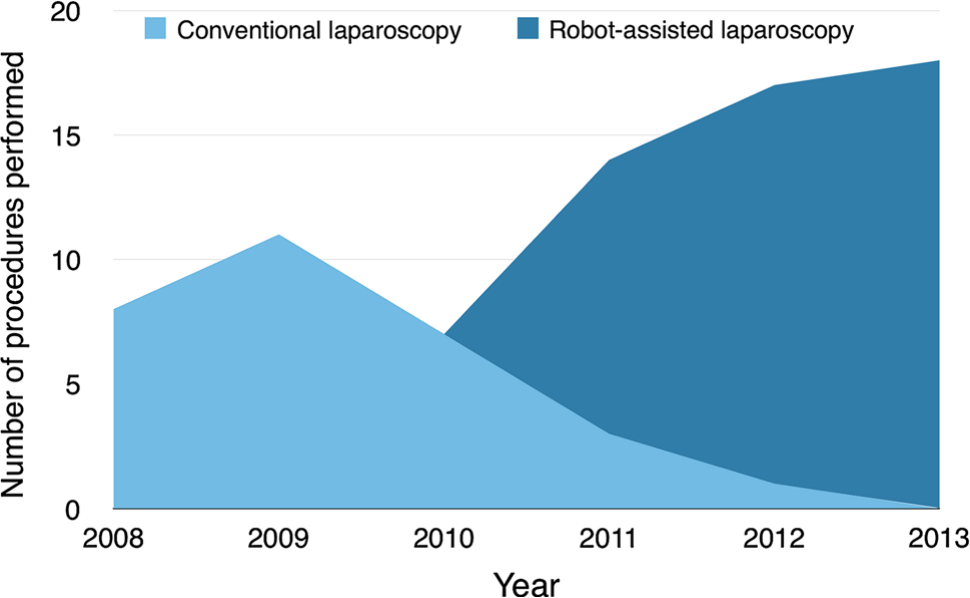
Distribution of redo antireflux interventions

From January 2008 to December 2013, 75 patients underwent a total of 83 redo procedures. The conventional laparoscopic redo surgery group consisted of 30 patients who underwent their intervention between January 2008 and July 2012. The robot-assisted redo surgery group consisted of 45 patients who underwent their intervention between March 2011 and December 2013 (Fig. 1). Some patients underwent conventional laparoscopic redo surgery after introduction of the da Vinci robot due to the robotic system being unavailable at the time of surgery. Eight patients (4 from both groups) underwent a second redo procedure. This was performed by conventional laparoscopic surgery or robot-assisted surgery in 2 and 6 patients, respectively.

Demographics for both groups are summarized in Table 1. There were no significant differences in age, gender, preoperative symptoms or comorbidity between the two groups. However, the number of patients in the robot-assisted laparoscopy group with a previous antireflux procedure via laparotomy was significantly higher than in the conventional laparoscopy group (9/45 vs. 1/30, *p* = 0.038). The majority of patients had previously undergone one antireflux procedure. The main indication for redo surgery was dysphagia (20/75), pyrosis (13/75), regurgitation (5/75) or a combination of these symptoms (37/75). The mean time to redo hiatal hernia and antireflux surgery was 1.9 (0.9–3.2) and 2.0 (1.2–5.4) years after the previous antireflux procedure for the conventional and robot-assisted groups, respectively. The details of the redo hiatal hernia and antireflux surgical procedures are presented in Table 2.

**Table 1:** Preoperative patient characteristics and complaints

	Conventional laparoscopy *n* = 30	Robot-assisted laparoscopy *n* = 45
Age in years, mean	57 (53–62)	56 (51–60)
Gender		
Male	11	12
Female	19	33
Number of previous reflux procedures		
One	27	38
Two	3	7
One or more previous procedures via laparotomy	1*	9*
Pattern of complaints		
Pyrosis	5	8
Regurgitation	2	3
Dysphagia	10	10
Pyrosis and regurgitation	5	8
Regurgitation and dysphagia	2	2
Pyrosis and dysphagia	3	4
Pyrosis, regurgitation and dysphagia	2	9
Charlson Comorbidity Score		
0	24	30
1	2	8
2	1	7
Number of days since previous surgery in days, median	702 (319–1286)	742 (442–1973)

Values are expressed as median (interquartile range), mean (95 % CI) or number of patients

* *p* = 0.038 (Fischer’s exact test)

**Table 2:** Details of redo antireflux procedures

	Conventional laparoscopy *n *= 30	Robot-assisted laparoscopy *n * = 45	*p* value
Duration of surgery in minutes, median	95 (90–115)	120 (110–120)	0.098
Hiatal herniation			
No herniation	12	9	0.040
Type I—sliding hiatal hernia	10	20	
Type II—paraesophageal hiatal hernia	6	3	
Type III—mixed type hiatal hernia	1	7	
Type IV—giant hiatal hernia with intrathoracic stomach	1	6	
Performed procedure (at the crus)			
None	8	4	0.064
Hiatal hernia repair	17	36	
Widening of the hiatus	4	4	
Performed procedure (at the fundus)			
None	0	4	0.020
Takedown of previous fundoplication and re-fundoplication	25	25	
No previous fundoplication to take down, just fundoplication	4	15	
Fundoplication type			
None	1	2	0.167
Nissen	6	4	
Toupet	20	27	
Dor	2	12	
Use of mesh			
None	21	18	<0.001
Mesh	8	27	

Values are expressed as mean (95 % CI) or number of patients

Most patients received a Toupet fundoplication (47/75), 14 an anterior fundoplication and 10 a Nissen fundoplication. In three cases, the previous fundoplication was taken down without refundoplication. There was no significant difference in mean operating time or type of surgical procedure performed. The use of a polypropylene mesh differed. In the conventional group 8 out of 30 of patients underwent mesh enforcement of the crus, compared to 27 out of 45 in the robot-assisted group.

There was no in-hospital or early postoperative mortality. 26 out of 30 conventional laparoscopic redo procedures and 38 out of 45 in the robot-assisted group were performed without any complications (Table 3). In the robot-assisted group there were significantly fewer conversions to laparotomy than in the laparoscopic group (1/45 vs. 5/30, *p* = 0.035). All conversions were due to the inability to safely proceed due to adhesions, perforation or inability to recognize anatomy. During the dissection, two patients had a minor bleeding which was easily managed by using an electrothermal system (Ligasure). During hernia sac removal, a pleural defect occurred in two patients, one of which required a chest tube. A total of four gastric perforations and three esophageal perforations were seen. All of them could be managed laparoscopically.

**Table 3:** Surgical complications

	Conventional laparoscopy *n * = 30	Robot-assisted laparoscopy *n * = 45
Surgical mortality	0	0
Major complications		
Pleural defect	0	2
Esophagus perforation	2	1
Minor complications		
Bleeding	0	2
Gastric perforation	2	2

Values are expressed as number of patients

Median hospital stay was shorter in the robot-assisted group by 1 day [3 (2–5) vs 4 (3–7), *p* = 0.042]. Two patients from the conventional group had a severe complication for which ICU admission was required. Both developed a mediastinitis, both most likely due to an unrecognized esophagus perforation by dissection or thermal injury. The latter patient required three thoracotomies. Both patients survived. Six patients had minor complications requiring surgical intervention (fascial dehiscence) or medical treatment (delirium, asthma exacerbation, opiate overdosage). There was one wound infection in the conventional group.

Median follow-up time was 10 (3–24) months and 3 (1–11) months for the conventional and robot-assisted groups, respectively. 54 % of all patients in both groups reported to have no complaints or easily manageable complaints (Table 4). There was no significant difference between both groups with regard to postoperative symptoms.

**Table 4:** Symptomatic outcome

	Conventional laparoscopy *n * = 30	Robot-assisted laparoscopy *n * = 45	*p* value
Follow-up time in days	309 (94–723)	87 (38–326)	0.007
Pattern of complaints			
No complaints or easily manageable	14	22	0.653
Pyrosis	7**	5*	
Regurgitation	0	1	
Dysphagia	2*	5**	
Pyrosis and regurgitation	1	0	
Regurgitation and dysphagia	0	0	
Pyrosis and dysphagia	1*	1*	
Pyrosis, regurgitation and dysphagia	0	0	
Delayed gastric emptying	3	3	
Chronic diarrhea	0	2	

Values are expressed as median (interquartile range) or number of patients. Each * represents one patient that required another redo antireflux surgical procedure to alleviate symptoms

In the conventional laparoscopic group, four patients required another redo hiatal hernia and antireflux procedure. All patients had recurrent GERD symptoms, two due to migration of the fundoplication, the others were without anatomical aberrations. Three patients had previously had a Toupet fundoplication which was taken down and a Nissen fundoplication made. The last patient had a Dor fundoplication with a sliding hernia which was corrected via cruroplasty. In the robot-assisted group 4 patients underwent a second redo procedure. Three had recurrent GERD symptoms, the remaining patient both dysphagia and pyrosis. All patients had migration of the fundoplication or recurrence of a previous para-esophageal herniation. They all had a Toupet fundoplication from the previous redo surgery. This was taken down and after cruroplasty a Dor fundoplication was made.

## Discussion

Since the introduction of robotic systems, surgeons have been endeavoring to determine the right indications to apply this complex technology. Several small randomized studies have been performed to evaluate its use with primary antireflux surgery, but failed to prove true superiority of the robotic system over conventional laparoscopic surgery [15, 17–19]. This study is the first to evaluate conventional laparoscopic versus robot-assisted laparoscopic redo antireflux surgery.

In this single-center observational case series, we found a significant reduction in the number of conversions in the robot-assisted redo hiatal hernia and antireflux group despite the fact that more patients underwent previous antireflux surgery via laparotomy. Also, hospital stay was reduced by 1 day while patients reported a similar symptomatic outcome.

Even though this study is of an observational design, we believe that any bias has been brought to a minimum as all procedures were performed by a single surgeon with extensive experience in laparoscopic primary and redo hiatal hernia and antireflux procedures and extensive previous robotic experience. This eliminated the learning curve in the conventional laparoscopic group and vastly reduced the robotic curve [20]. The results of this study, however, have to be interpreted with caution due to the methodological limitations associated with observational studies.

The documented average hospital stay for patients who underwent redo surgery is 5.5 days [10]. In our laparoscopic group we found a median stay of 4 days, in the robot group 3—a significant difference. If the patients with lengthy ICU admissions in the conventional laparoscopic group are omitted from statistical analysis, a significant difference in hospital stay remains (*p* = 0.034). The significant shorter hospital stay therefore cannot be explained by the cases with prolonged ICU admission, but has to be correlated to the reduced number of conversions in the robotic group.

Redo surgery for recurrent hiatal hernia or failed fundoplication is usually more demanding due to dense adhesions, changed anatomy and inability to find the correct planes for dissection. This leads to an increased complication rate and also a high conversion rate.

The most common complication during redo surgery is a perforation of the stomach or esophagus. In our series, surgery was complicated by perforation in seven (9 %) cases, a lower rate than reported in literature (13–14 %) [10, 21].

Known reasons for converting laparoscopic redo hiatal hernia and antireflux surgery are dense adhesions (80.2 %), severe intra-operative bleeding (15.5 %) or poor visualization (4.3 %) [10]. Robotic systems may offer improvement in both visualization of the operative field and technical feasibility when dissecting adhesions and taking down any previous fundoplications. Also, more patients who had previously undergone open (abdominal) antireflux surgery could be managed laparoscopically with the use of the robot.

With this study we have shown that operating these patients using a robot-enhanced minimal invasive technique is not only feasible but also safe. An argument often used in favor for the open approach, the fact that previous antireflux surgery was performed via laparotomy, may no longer be valid.

## Conclusion

Robotic support, when available, can be regarded beneficial in redo surgery for GERD and hiatal hernia. Results of this observational study suggest technical feasibility for minimal-invasive robot-assisted redo surgery after open primary antireflux surgery, a reduced number of conversions and shorter hospital stay.
